# Reirradiation for Recurrent Cervical Cancer Within the Previous Radiation Field Using a Bioabsorbable Spacer: A Case Report

**DOI:** 10.1111/jog.70225

**Published:** 2026-03-06

**Authors:** Yusuke Matoba, Yuriko Oomori, Ikuno Nishibuchi, Shinnosuke Uegami, Kosuke Nakamoto, Takuto Uyama, Katsuyuki Tomono, Suguru Nosaka, Hiroki Ohge, Shinya Takahashi, Yuji Murakami, Ken Yamaguchi, Kouji Banno

**Affiliations:** ^1^ Department of Obstetrics and Gynecology Hiroshima University Hospital Hiroshima Japan; ^2^ Department of Radiation Oncology, Graduate School of Biomedical and Health Sciences Hiroshima University Hiroshima Japan; ^3^ Department of Surgery, Graduate School of Biomedical and Health Sciences Hiroshima University Hiroshima Japan; ^4^ Department of Obstetrics and Gynecology, Graduate School of Biomedical Sciences Hiroshima University Hiroshima Japan; ^5^ Center of Maternal‐Fetal/Neonatal Medicine Hiroshima University Hospital Hiroshima Japan

**Keywords:** bioabsorbable spacer, cervical cancer, radiotherapy, recurrence, reirradiation

## Abstract

Reirradiation for cervical cancer recurrence in previously irradiated fields is challenging due to dose limitations. To our knowledge, this is the first case report describing successful reirradiation using a bioabsorbable spacer for vaginal cuff recurrence after initial concurrent chemoradiotherapy (CCRT) and hysterectomy. A 70‐year‐old woman with cervical cancer Stage IIIC2r initially received CCRT and chemotherapy. Three years later, uterine recurrence led to hysterectomy. Eighteen months post‐surgery, vaginal cuff recurrence was diagnosed by imaging and tumor biopsy. A bioabsorbable spacer was surgically placed around the vaginal cuff tumor via an open abdominal approach. Reirradiation (55 Gy in 22 fractions) was started 23 days postoperatively. Use of the spacer provided adequate dose reduction to the small bowel, sigmoid colon, and rectum, with the rectal D1cc reduced to 37.9 Gy. Mild paralytic ileus occurred, but resolved conservatively, and there were no severe complications. The patient remains disease‐free at 6 months post‐treatment. In this case, bioabsorbable spacer placement allowed safe reirradiation for cervical cancer vaginal cuff recurrence. This technique may represent a promising approach for selected patients with in‐field recurrent cervical cancer, although further accumulation of cases and longer follow‐up are required.

## 1 Introduction

Cervical cancer is a malignant disease affecting the female reproductive organs, with approximately 600 000 new cases annually worldwide [1]. Squamous cell carcinoma is the most common histological type, and its association with human papillomavirus (HPV) has been established; therefore, HPV vaccination is expected to decrease the incidence of cervical cancer [1]. Early‐stage cervical cancer is primarily treated with surgery, while advanced‐stage (Stage II or higher) cases typically receive radiotherapy or concurrent chemoradiotherapy (CCRT) using cytotoxic agents like cisplatin as initial treatment [2]. The recurrence rate for early‐stage cervical cancer (FIGO stage IB‐IIA) after initial treatment ranges from 11%–22%, while the range for advanced cervical cancer (IIB‐IVA) is 28%–64% depending on treatment modalities and other risk factors [3, 4]. Local recurrence is common in early‐stage cervical cancer, whereas advanced cases often present with both local and distant metastases [3].

Radiotherapy is a treatment option for local recurrence in patients with cervical cancer without prior radiation therapy [5]. However, reirradiation, especially for local recurrence within a previously irradiated field, poses significant challenges due to the dose limitations of normal tissues [5, 6]. The incidence of grade 2 late toxicities increases when the cumulative D2cc equivalent dose in 2‐Gy fractions (EQD2), derived from the combination of external beam radiation therapy (EBRT) and brachytherapy, exceeds 80 Gy to the bladder, 65 Gy to the rectum, 70 Gy to the sigmoid colon, and 70 Gy to the small bowel [7]. Given that initial cervical cancer treatment often involves 45–50 Gy to the whole pelvis with EBRT and 18–24 Gy in 3–4 fractions of brachytherapy, reirradiation within the same field is generally avoided [5].

There have been recent advances in radiation techniques, such as intensity‐modulated radiation therapy (IMRT), volumetric modulated arc therapy (VMAT), and image‐guided brachytherapy (IGBT), and concurrently, methods have been developed to mitigate radiation effects on normal tissues by inserting bioabsorbable spacers between normal and tumor tissues. These spacers have been reported to improve radiotherapy safety in other cancer types, including prostate and liver cancers, by creating physical distance between the target lesion and critical organs, thereby improving dose distribution and enabling safe dose escalation [8, 9]. Despite these advances, to our knowledge, there are no published reports on the use of bioabsorbable spacers for reirradiation of in‐field recurrence of cervical cancer.

Here, we report a case of a 65‐year‐old woman with in‐field vaginal cuff recurrence of cervical cancer after initial CCRT. This report is the first, to our knowledge, to show that placement of a bioabsorbable spacer between the tumor and surrounding organs can facilitate safe readministration of concurrent chemoradiotherapy. Therefore, this case illustrates a potentially novel approach for managing challenging cases with recurrence of cervical cancer within a previously irradiated field.

## 2 Case Presentation

**FIGURE 1:**
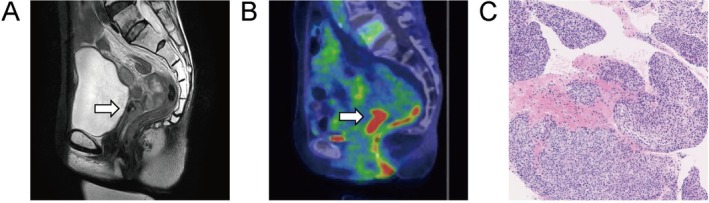
Findings for recurrence. (A) MRI (T2‐weighted image) showed a recurrent tumor of about 2 cm at the vaginal cuff after hysterectomy (arrow). (B) PET‐CT showed uptake in the tumor at the same location of the vaginal cuff identified on MRI (arrow). (C) Pathological findings from a transvaginal tumor biopsy indicated squamous cell carcinoma, similar to the primary lesion.

The patient was a 70‐year‐old woman with an Eastern Cooperative Oncology Group (ECOG) Performance Status (PS) of 0, a body mass index (BMI) of 16.9 kg/m^2^, and no significant comorbidities. She had a history of Stage IIIC2r cervical cancer that was initially treated with CCRT and later with a hysterectomy for uterine recurrence. She first received external beam radiotherapy to the whole pelvis (55 Gy in 30 fractions), followed by high‐dose‐rate (HDR) brachytherapy (RALS). The brachytherapy delivered 15.9 Gy in 3 fractions to 90% of the clinical target volume (CTV D90), with a corresponding dose of 10.1 Gy in 3 fractions to Point A. She presented 1.5 years post‐surgery with a second recurrence. A mass appeared at the vaginal cuff on CT, with uptake with SUVmax 17.9 on PET‐CT. Tumor biopsy revealed squamous cell carcinoma, which was diagnosed as cervical cancer recurrence (Figure 1). Surgical options, including local resection or pelvic exenteration, were considered to be viable for this recurrence. However, given the patient's age and history of repeated pelvic recurrences, the multidisciplinary team concluded that even with pelvic exenteration, adjuvant therapy, including reirradiation, would likely still be required. Therefore, the team opted for a less invasive, although still surgical, approach: reirradiation after surgical placement of a bioabsorbable spacer (NESKEEP, Alfresa Pharma Corp., Osaka, Japan) between the tumor and rectum. This device is a single‐layer bioabsorbable spacer composed of polyglycolic acid. A product of 10‐mm thickness was used. This spacer can maintain about 85% of its thickness at 2 months and is almost completely absorbed by about 8 months. Because about 5 years had passed since the previous chemotherapy, the tumor was also expected to be platinum‐sensitive. Therefore, we decided to administer chemotherapy concurrently with radiotherapy, with careful monitoring for adverse events.

**FIGURE 2:**
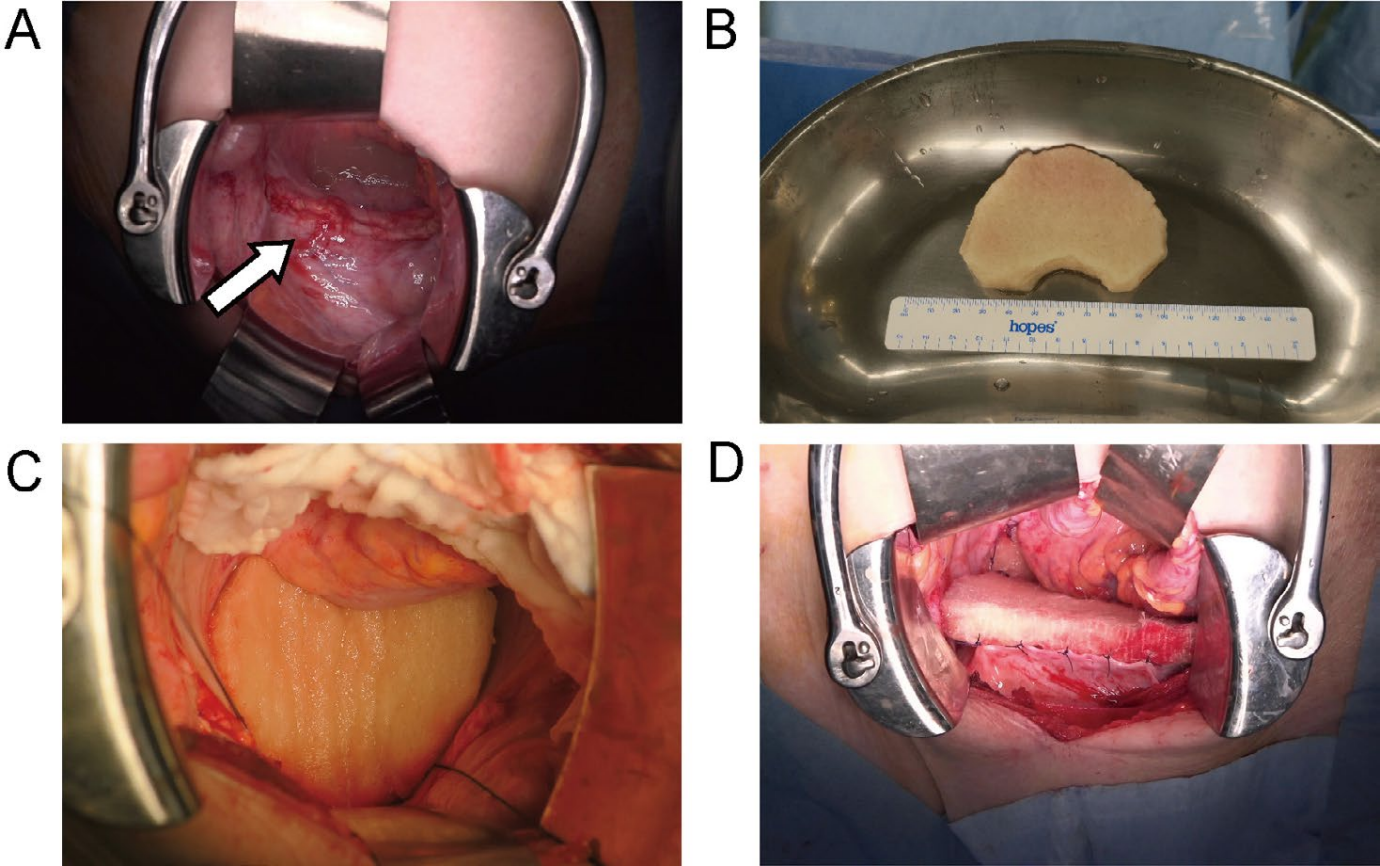
Surgical findings during bioabsorbable spacer placement (cranial side at top). (A) Vaginal cuff recurrent tumor (arrow). (B) Bioabsorbable spacer shaped to fit the pelvic cavity. (C) After placement of the bioabsorbable spacer. (D) After suture fixation of the bioabsorbable spacer to the pelvic wall.

During open surgery, the recurrent tumor at the vaginal cuff was identified (Figure 2A) and a space was created by dissecting the rectum away from the tumor. The spacer material was first thoroughly soaked in saline to prevent air entrapment, which could compromise its dosimetric properties. It was then cut with scissors to fit the pelvic space, forming an approximately 8 × 5 cm shape. To create sufficient distance from organs at risk (OARs), two layers of the spacer were stacked between the tumor and rectum. A single layer was placed on the bladder side, where the distance was already macroscopically sufficient. Fixation was performed using 3–0 braided absorbable sutures, with approximately 10 stitches for each spacer, securing them firmly to the pelvic wall and surrounding tissues (Figure 2B–D). Peritoneal cytology was negative.

**FIGURE 3:**
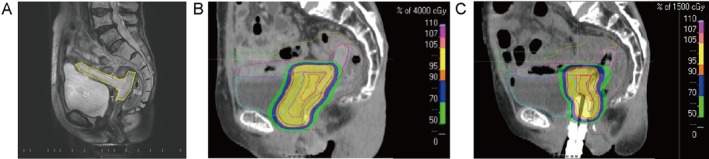
Treatment planning after bioabsorbable spacer placement. (A) MRI (sagittal T2‐weighted image) showed that the bioabsorbable spacer (yellow dotted line) had created distance between the vaginal recurrent tumor (T) and rectum (Ra). The distance created by the spacer insertion between the tumor and the rectum was 13 mm. Bl indicates the bladder. (B, C) Dose distribution in radiation therapy planning. (B) Dose distribution up to 40 Gy, (C) Dose distribution after 40 Gy.

The position of the spacer was confirmed on postoperative MRI. The distance between the tumor and the rectum was 13 mm. Reirradiation using VMAT was initiated 60 months after completion of the initial radiotherapy, delivering a total dose of 55 Gy in 22 fractions with concurrent weekly nedaplatin at 30 mg/m^2^. After 40 Gy, a vaginal cylinder was used part way through treatment to further protect the bladder (Figure 3). The CTV included the posterior vaginal wall and 14 mm caudal to the gross tumor volume. A planning target volume margin of 10 mm was applied in the caudal direction and 8 mm in all other directions. To maintain a consistent bladder volume, a urethral balloon was placed with 120 mL of saline instilled before each treatment. Positional verification using cone‐beam computed tomography was performed at each session to confirm that there was no displacement between the spacer and target. The only complication was a temporary paralytic ileus that resolved with fasting. However, administration of chemotherapy was discontinued due to formation of the paralytic ileus.

Irradiation doses to OARs are shown in Table S1. To estimate the doses in the absence of a spacer, three spacer regions were evaluated separately: between the small bowel and the target (Spacer1_bowel), between the sigmoid colon and the target (Spacer2_s‐colon), and between the rectum and the target (Spacer3_rectum). The results show that spacer placement reduced the doses to all OARs. The spacer was irradiated with approximately the full prescribed dose of 55 Gy. In the absence of spacer placement, it is assumed that the cumulative dose to each OAR, including the prior irradiation, would have exceeded 100 Gy. The patient completed treatment without any other adverse events. A CT scan 2 months later showed tumor reduction, with the bioabsorbable spacer retaining approximately 80% of its thickness in nearly the same position as at insertion. At 6 months post‐irradiation, the patient was progressing well. Cytology of the vaginal cuff showed no malignant findings, and imaging studies revealed no signs of recurrence. At about 7 months after spacer placement, the spacer was almost indiscernible.

## 3 Discussion

This case shows that placing a bioabsorbable spacer can facilitate safe reirradiation for in‐field recurrence of cervical cancer without severe adverse events. Radiotherapy is a standard primary treatment for cervical cancer Stage IB3 or higher [2], and recurrence within the original radiation field is a common challenge [3]. The success of using a spacer in this case suggests a potential shift in treatment strategies for similar future cases. Previous reports in gynecologic oncology have described transvaginal injection of a hydrogel spacer to enable reirradiation for in‐field recurrence [10], or use of an omental flap transposition to protect OARs during reirradiation [11]. However, to our knowledge, this is the first report of surgical placement of a solid bioabsorbable spacer for reirradiation in a patient with in‐field recurrence of cervical cancer.

Treating in‐field recurrence of cervical cancer is notoriously difficult. For solitary recurrence, surgical options such as local resection or pelvic exenteration may be considered, but these are associated with significant morbidity and difficulty achieving complete resection [12]. Reirradiation also carries a high risk of exceeding the radiation tolerance doses of normal organs, which can lead to severe complications. Thus, even with advanced radiation techniques [6, 13, 14], reirradiation is generally not recommended. Pelvic organs like the bowel, rectum, and bladder are highly radiosensitive, and exceeding their cumulative dose tolerance increases the risk of irreversible late complications such as bowel stenosis, perforation, bladder contracture, and hematuria [15].

Bioabsorbable spacers have recently been used in prostate and liver cancers to improve radiotherapy safety by reducing the dose to surrounding organs [8, 9]. These spacers create physical distance between the target lesion and critical organs, which improves the dose distribution to normal tissues and allows for safe dose escalation. The current case suggests that this principle is also applicable to reirradiation for locally recurrent cervical cancer. In the patient's radiation treatment plan, the spacer reduced the radiation dose delivered to the small bowel, sigmoid colon, and anterior rectal wall, enabling safe reirradiation. The absence of severe adverse events, such as rectal or bladder complications, during treatment and follow‐up shows the effectiveness of this strategy.

In addition to radiotherapy, we planned concurrent chemotherapy in our case. There is currently no established evidence for CCRT for in‐field recurrence after initial CCRT, although the usefulness of reirradiation alone has been reported with recent advances in radiotherapy techniques [13]. In our patient, the recurrence occurred 4 years after the initial CCRT and one and a half years after surgery for the first recurrence. We therefore hypothesized that the tumor might be platinum‐sensitive, and decided to administer concurrent chemotherapy while monitoring for adverse events. However, the chemotherapy was discontinued after only one cycle due to the development of paralytic ileus. Consequently, the contribution of chemotherapy to the outcome is unclear. Further investigation is needed to determine whether adding chemotherapy to reirradiation improves the prognosis for in‐field cervical cancer recurrence. Cervical cancer treatment is advancing, from prevention with HPV vaccine [1] to the use of high‐precision radiotherapy techniques like IGBT, IMRT, and VMAT for initial treatment [16] and immune checkpoint inhibitors and molecular targeted therapies for both primary and recurrent disease [17, 18]. Use of bioabsorbable spacers for reirradiation may be another useful option for recurrent cervical cancer, particularly for in‐field recurrence.

We note that this treatment approach has several limitations. Careful patient selection is critical and ideal candidates are likely to be those with limited local recurrence, good performance status, and no distant metastasis. Spacer placement is likely to be beneficial only when the recurrence is solitary or confined to a localized area. For cases involving extensive disseminated lesions, systemic therapy remains the preferred option. Additionally, placing the spacer requires a surgical procedure, which necessitates a thorough preoperative evaluation of overall health, surgical risks, and the feasibility of the placement itself. The effectiveness of the treatment is also highly dependent on the final position and shape of the spacer, requiring close collaboration between radiation oncologists and surgeons. Finally, a significant limitation is the short follow‐up duration of this single case. At 6 months post‐reirradiation, the patient is currently alive with no evidence of recurrence or severe complications. However, longer‐term follow‐up is needed to assess long‐term oncological control and the potential for future recurrence. Moreover, continued monitoring is crucial for detecting late radiation‐related toxicities, as these often manifest more than 3 months after completion of radiotherapy [19]. Further follow‐up for recurrence, including cytologic examinations and imaging studies, is scheduled at approximately three‐month intervals.

In conclusion, this case demonstrates that reirradiation for in‐field cervical cancer recurrence is feasible and potentially beneficial with placement of a bioabsorbable spacer. Accumulation of more cases, establishment of criteria for patient selection, standardization of surgical techniques, and evaluation of long‐term prognosis are important next steps to validate this method as a potential treatment option.

## Author Contributions

**Yusuke Matoba:** conceptualization, investigation, writing – original draft, writing – review and editing, data curation, software, visualization, project administration. **Yuriko Oomori:** conceptualization, methodology, resources, data curation, investigation, writing – review and editing. **Ikuno Nishibuchi:** conceptualization, methodology, data curation, investigation, writing – review and editing. **Shinnosuke Uegami:** conceptualization, methodology, data curation, investigation, writing – review and editing. **Kosuke Nakamoto:** investigation, resources, writing – review and editing. **Takuto Uyama:** investigation, resources, writing – review and editing. **Katsuyuki Tomono:** investigation, resources, writing – review and editing. **Suguru Nosaka:** investigation, resources, writing – review and editing. **Hiroki Ohge:** investigation, resources, writing – review and editing, supervision. **Shinya Takahashi:** investigation, resources, writing – review and editing, supervision. **Yuji Murakami:** investigation, resources, writing – review and editing, supervision. **Ken Yamaguchi:** investigation, resources, writing – review and editing, project administration, supervision. **Kouji Banno:** investigation, resources, writing – review and editing, funding acquisition, supervision.

## Disclosure

The authors have nothing to report.

## Ethics Statement

The study was approved by the Institutional Ethics Committee of Hiroshima University Hospital (Approval No. E2025‐0009).

## Consent

Written informed consent was obtained from the patient for publication of this case report and accompanying images.

## Conflicts of Interest

Dr. Ken Yamaguchi is a member of the Editorial Board of the Journal of Obstetrics and Gynecology Research. To minimize bias, Dr. Yamaguchi was excluded from all editorial decision‐making related to the acceptance of this article for publication.

## Supporting information


**Table S1:** Irradiation doses to organs at risk.

## Data Availability

The data that supports the findings of this study are available in the Supporting Information of this article.
