# Supplementary material for: Reirradiation for Recurrent Cervical Cancer Within the Previous Radiation Field Using a Bioabsorbable Spacer: A Case Report
Source: J Obstet Gynaecol Res. 2026 Mar 6;52(3):e70225. doi: 10.1111/jog.70225 (PMC12965828; doi:10.1111/jog.70225)
Supplement: Supplementary file 1 — Table S1: Irradiation doses to organs at risk. [file JOG-52-0-s001.docx]

| Organ | Dmax | D1cc | D2cc | D10cc |
| --- | --- | --- | --- | --- |
| Small bowel | 16.1 Gy | 7.2 Gy | 6.1 Gy | 3.5 Gy |
| Sigmoid colon | 34.5 Gy | 29.4 Gy | 20.5 Gy | 6.7 Gy |
| Whole rectum | 50.3 Gy | 37.9 Gy | 34.6 Gy | 24.3 Gy |
| Rectum with spacer* | 43.2 Gy | 27.4 Gy | 23,2 Gy | 10.2 Gy |
| Bladder | 55.6 Gy | 55.3 Gy | 55.2 Gy | 51.6 Gy |
| Spacer1 (bowel) | 55.4 Gy | 26.7 Gy | 22.3 Gy | 11.2 Gy |
| Spacer2 (s-colon) | 55.9 Gy | 55.5 Gy | 55.3 Gy | 52.2 Gy |
| Spacer3 (rectum) | 55.4 Gy | 55.1 Gy | 55.0 Gy | 52.3 Gy |

Supplementary Table 1: Irradiation doses to organs at risk

* Rectal region with the spacer
